# Antibody indices of infectious pathogens from serum and cerebrospinal fluid in patients with schizophrenia spectrum disorders

**DOI:** 10.1186/s12987-022-00355-7

**Published:** 2022-07-29

**Authors:** Kimon Runge, Agnes Balla, Bernd L. Fiebich, Simon J. Maier, Benjamin Pankratz, Andrea Schlump, Kathrin Nickel, Rick Dersch, Katharina Domschke, Ludger Tebartz van Elst, Dominique Endres

**Affiliations:** 1 Department of Psychiatry and Psychotherapy, Medical Center – University of Freiburg, Faculty of Medicine, University of Freiburg, Freiburg, Germany; 2 Clinic of Neurology and Neurophysiology, Medical Center – University of Freiburg, Faculty of Medicine, University of Freiburg, Freiburg, Germany; 3 Center for Basics in Neuromodulation, Faculty of Medicine, University of Freiburg, Freiburg, Germany

**Keywords:** EBV, CMV, HSV, Toxoplasmosis, Antibody index, Schizophrenia, Psychosis, SSD, Autoimmune

## Abstract

**Introduction:**

Infectious and immunological theories of schizophrenia have been discussed for over a century. Contradictory results for infectious agents in association with schizophrenia spectrum disorders (SSDs) were reported. The rationale of this study was to investigate intrathecal antibody synthesis of the most frequently discussed neurotropic pathogens using a pathogen-specific antibody index (AI) in patients with SSD in comparison to controls.

**Methods:**

In 100 patients with SSD and 39 mentally healthy controls with idiopathic intracranial hypertension (IIH), antibodies against the herpesviruses EBV, CMV, and HSV 1/2 as well as the protozoan Toxoplasma gondii, were measured in paired cerebrospinal fluid (CSF) and serum samples with ELISA-kits. From these antibody concentrations the pathogen-specific AIs were determined with the assumption of intrathecal antibody synthesis at values > 1.5.

**Results:**

No significant difference was detected in the number of SSD patients with elevated pathogen-specific AI compared to the control group. In a subgroup analysis, a significantly higher EBV AI was observed in the group of patients with chronic SSD compared to patients with first-time SSD diagnosis (p = 0.003). In addition, two identified outlier EBV patients showed evidence for polyspecific immune reactions (with more than one increased AI).

**Conclusions:**

Evidence for the role of intrathecal EBV antibody synthesis was found in patients with chronic SSD compared to those first diagnosed. Apart from a possible infectious factor in SSD pathophysiology, the evidence for polyspecific immune response in outlier patients may also suggest the involvement of further immunological processes in a small subgroup of SSD patients.

**Supplementary Information:**

The online version contains supplementary material available at 10.1186/s12987-022-00355-7.

## Introduction

Schizophrenia is a severely disabling mental illness [29]. Due to the heterogeneous presentation of clinical manifestations and biological parameters, one can assume various interrelated etiologies of schizophrenia spectrum disorders (SSDs) [49]. Rarely, the etiology can be identified as in autoimmune psychosis caused by well-characterized antibodies like those against the anti-N-Methyl-d-aspartate receptor (NMDA-R) [35]. In spite of intensive research, other causes or underlying pathomechanisms remain widely elusive. In addition to a distinct genetic component [42], infectious [5] and immunological causes [15, 39] are frequently discussed. Among neurotropic infectious agents that have been associated most consistently with schizophrenia are Toxoplasma gondii as well as herpesviridae of the herpes simplex group (HSV1/2), cytomegalovirus (CMV), and the Epstein-Barr virus (EBV) [5, 63].

To test for exposure to infectious agents in patients with mental illnesses, various methods measuring antigens, antibodies, and nucleic acids (DNA/RNA) have been applied with mixed results [4]. Regarding antibody measurements, pathogen-specific antibodies were often detected in serum, rarely in cerebrospinal fluid (CSF), and seldom in both. Of the latter, titers have been mainly measured, of which a CSF/serum quotient was calculated. However, analyses of CSF/serum titer quotients lack sensitivity in detection of intrathecal antibody synthesis [38, 47]. This, in contrast, can be achieved with calculation of the specific antibody index (AI) in paired CSF/serum pathogen specific antibody concentrations, which allows for the identification of pathologically elevated brain-derived antibody fractions [38]. The potential elevation of AIs without further traces of infections, is a promising investigative marker for the association of infections with SSDs [38, 47]. Furthermore, several elevated AIs as polyspecific immune reactions can indicate immunological processes in the central nervous system (CNS; this has been reported in multiple sclerosis for MRZ reaction) [20].

Nonetheless, no study could be identified investigating intrathecal antibody synthesis against T. gondii and the Herpesviridae HSV1/2, CMV, and EBV using pathogen-specific AIs in SSDs. Therefore, the rationale of this study was to investigate the fractions of brain-derived antibody synthesis of specific antibodies using AIs in patients with SSD and compare them with a mentally healthy control group to test the association of the four aforementioned infectious agents with SSDs. Furthermore, several elevated AIs should provide insight into polyspecific immune reactions as possible inflammatory signs of the CNS, investigating immunological causes of schizophrenia.

## Methods

Ethical approval for the current study was provided by the local ethics committee in the context of a larger retrospective research project (Medical Faculty of the University of Freiburg, EK-Fr 609/14). Patients with SSDs gave their written informed consent before lumbar puncture. The control patients were contacted retrospectively and asked for their consent to use the residual serum/CSF material for research purposes.

### Study sample

In the patient group, 100 consecutive patients with SSD treated as inpatients in the Department of Psychiatry and Psychotherapy of the Medical Center of the University of Freiburg were included. Diagnoses and comorbidities were established by experienced senior consultant psychiatrists according to ICD-10 criteria and extracted for this study through chart review. Exclusion criteria were defined as known substance use disorders and immunological disorders known for brain involvement. All patients were offered a magnetic resonance imaging (MRI) scan of the brain and an electroencephalography (EEG) as part of the routine workup. During the admission routine, comprehensive demographic and clinical data are collected. In addition, psychometric scales, such as the Global Assessment of Functioning (GAF) [2], Clinical Global Impression (CGI) [41], and psychopathological scores based on guidelines published by the German Association for Methodology and Documentation in Psychiatry (AMDP) [9] were assessed.

A mentally healthy control group consisted of 39 patients with idiopathic intracranial hypertension (IIH). The exclusion criteria for the control group were as follows: secondary forms of intracranial hypertension and psychiatric or other neurological disorders (except headaches). The control group was already published in earlier articles [27, 31, 39, 40].

### Antibody index

AI enables the detection of the intrathecal synthesis of a pathogen-specific antibody by distinguishing between antibodies originating in the brain and antibodies that passively cross the blood-CSF barrier (BCSFB) [38, 47]. This is mathematically possible by relating the specific CSF/serum antibody quotient (Q_spec_) to the CSF/serum quotient of total IgG antibodies (Q_IgG_; hence, AI = Q_spec_/Q_IgG_) [38]. However, because polyspecific increased intrathecal IgG production would lead to an underestimation of pathogen-specific antibodies, AI is calculated in reference to Q_lim_ if the total IgG exceeds Q_lim_: AI = Q_spec_/ Q_lim_ (if Q_IgG_ > Q_lim_) [38]. All IgG antibody measurements for EBV capsid antigen, HSV1/2, CMV, and T. gondii in serum and CSF were performed using commercially available enzyme-linked immunosorbent assay (ELISA) kits, according to the manufacturer’s instructions (Euroimmun®, Lübeck, Germany). Total IgG was determined nephelometrically using an Atellica NEPH 630 (Siemens Healthineers^®^, Erlangen, Germany) as specified by the manufacturer. Paired serum and CSF samples from the same day were measured consecutively on the same ELISA plate, side by side, to avoid inter-assay variation. ELISA standard curves for all parameters ranged from 5–100 Units (U; 230 in the case of HSV), so that a cut-off value of < 5 U was defined, at which the measurements were not considered positive. AI was calculated only if measurable antibody concentrations were found in CSF and serum. An AI between 0.7–1.5 was considered normal, and all values above this were considered positive for intrathecal antibody synthesis. All AIs below 0.7 were measured again and checked for plausibility. Since the ELISA kits were optimized for calculating AIs, group comparisons of the serum/CSF concentrations are possible, but no direct statement on total IgG values.

### CSF routine parameters and anti-neuronal antibodies

Routine CSF analyses and testing for anti-neuronal antibodies were conducted in the CSF laboratory of the Clinic of Neurology and Neurophysiology at the University Hospital Freiburg, which was described in earlier publications [15, 20]. For CSF antibodies against neuronal cell surface antigens (NMDA-R, LGI1, etc.), fixed cell-based assays were performed by Euroimmun® (Lübeck, Germany). For serum autoantibodies against paraneoplastic intracellular antigens (Yo, Hu, etc.) immunoblots by Ravo Diagnostika^®^ (Freiburg, Germany) were performed [15]. The remaining CSF was preserved at − 80 °C after routine analysis for further analysis.

### Statistical analyses

Statistical analyses of the acquired data were performed using the SPSS Version 27 (IBM, Armonk, USA). Group differences of patients and controls as well as subanalyses of patient subgroups for categorical variables (e.g., number of increased AIs) were statistically assessed by Chi-squared tests. For continuous variables (e.g., antibody concentrations), normality, assessed by the Shapiro–Wilk test, could only be assumed for a few variables. Therefore, a nonparametric Mann–Whitney U test for independent samples was conducted for all continuous variables. Secondary analysis with correction of sex was performed for continuous variables with an analysis of covariance (ANCOVA) and for categorical variables with binary logistic regression. Spearman’s rank correlation coefficient was used for the correlation between AIs and CSF routine parameters, age, psychometric scales, the number of suicide attempts and previous inpatient stays. Due to the descriptive nature of our study and the explorative nature of the subanalyses no correction for multiple testing was conducted. In all statistical analyses, a p-value lower than 0.05 was presumed statistically significant. All patients with antibody indices higher than two standard deviations above the mean value of the corresponding study group were characterized in more detail. Data visualization was realized with R package ggplot2 [37, 57] and Adobe Illustrator (Adobe Inc., San José, CA).

## Results

### Demographic data

In 100 patients with SSD and 39 controls, no significant difference in age (z = − 0.516, p = 0.606) could be identified. Predominantly, women were included in the patient group (60% women vs. 40% men) as well as the control group (85% women vs. 15% men), with a significant sex difference between the two groups (chi^2^ = 7.678, p = 0.006). When examining the patient group with SSD, paranoid schizophrenia (56%) with chronic or recurrent courses (58%) was mainly diagnosed. At the time of lumbar puncture, only three patients (3%) did not receive any psychiatric medication. All clinical and demographic data are presented in Additional file 2: Table S1.

### Pathogen specific antibody index

**Fig. 1:**
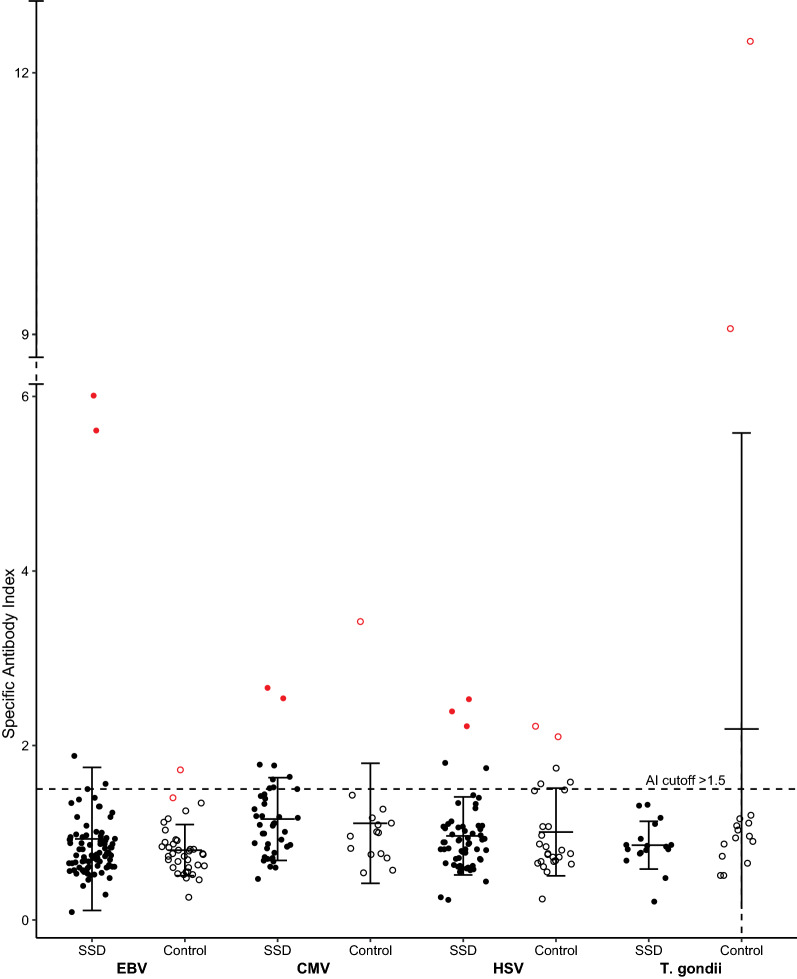
Specific antibody indices of the pathogens EBV, CMV, HSV and T. gondii in patients with schizophrenia spectrum disorder and controls with idiopathic intracranial hypertension. Error bars indicate mean value ± one standard deviation. All datapoints in red indicate outliers of more than 2 standard deviations over mean value (outlier patients are presented in detail in Sect. 3.6). Abbreviations: AI = Antibody Index, SSD = Schizophrenia spectrum disorder, EBV = Epstein–Barr virus, CMV = Human cytomegalovirus, HSV = Herpes simplex virus, T. gondii = Toxoplasma gondii

Several patients with elevated pathogen-specific AIs, indicating intrathecal IgG synthesis, were identified in the patient and control groups (Table 1 and Fig. 1). When comparing the number of patients with elevated AIs between SSD patients and controls, no significant differences were observed for any of the following pathogens: EBV, CMV, HSV, and T. gondii. For T. gondii, there is even a trend of frequently increased AIs in the control group (p = 0.061), and it is the only pathogen for which no elevated AI was found in the SSD group. There was also no significant difference in the number of patients with more than one elevated AI (p = 0.451). When applying higher thresholds for elevated AIs of more than two [10, 21], the group comparison of the number of patients with elevated AIs remained statistically insignificant. In a secondary analysis with correction for sex, the differences remained insignificant, and the tendency for an increased number of elevated T. gondii AIs in IIH controls could no longer be detected (Wald < 0.001, p = 0.999).

**Table 1:** Number of participants with elevated pathogen specific antibody indices (AIs)

	Elevated AIs in SSD patients (number of measurable AIs)	Elevated AIs in IIH controls (number of measurable AIs)	Statistics
EBV	4 (n = 84)	1 (n = 37)	Chi^2^ = 0.275, p = 0.600
CMV	8 (n = 31)	1 (n = 15)	Chi^2^ = 1.343, p = 0.246
HSV	5 (n = 59)	5 (n = 26)	Chi^2^ = 2.011, p = 0.156
T. gondii	0 (n = 17)	3 (n = 16)	Chi^2^ = 3.506, p = 0.061
Number of elevated AIs			Chi^2^ = 2.942, p = 0.451
0	85 (85%)	32 (82%)	
1	13 (13%)	5 (13%)	
2	2 (2%)	1 (3%)	
3	0 (0%)	1 (3%)	

*AI* antibody index, *SSD* schizophrenia spectrum disorder, *IIH* idiopathic intracranial hypertension, *EBV* Epstein–Barr virus, *CMV* human cytomegalovirus, *HSV* herpes simplex virus, *T. gondii* Toxoplasma gondii

**Fig. 2:**
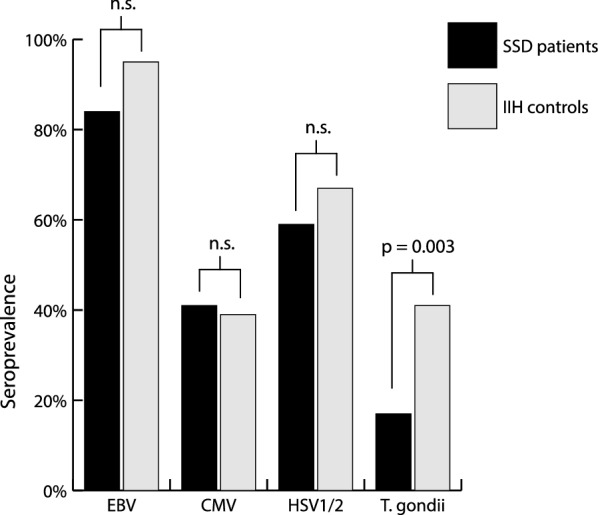
Seroprevalence of EBV, CMV, HSV and T. gondii antibodies in patients with schizophrenia spectrum disorder and controls with idiopathic intracranial hypertension. Abbreviations: SSD = Schizophrenia spectrum disorder, IIH = Idiopathic intracranial hypertension, n.s. = not significant, EBV = Epstein–Barr virus, CMV = Human cytomegalovirus, HSV = Herpes simplex virus, T. gondii = Toxoplasma gondii

When assessing the number of patients with measurable antibodies, the EBV showed the highest seroprevalence, with up to 95% in the control group (Fig. 2). The seroprevalence of herpesviruses is similar between the two study groups, while for T. gondii the fraction of seropositive SSD patients is with 17% less than half the fraction of patients in the control group with 41% (p = 0.003). After correction for sex, this effect remained significant (β = − 1.159, Wald = 7.171, p = 0.007).

Upon comparison of individual antibody concentrations in CSF and serum, a statistical difference between the CSF to serum ratios for EBV (p = 0.006) and CMV (p = 0.014) was striking (Table 2). However, when this quotient of pathogen-specific antibody concentrations from CSF and serum was related to the total antibody concentration and the blood-CSF function (i.e., the AI was calculated), no significant differences were observed. The results of the significantly higher CSF to serum ratios for EBV and CMV are further described and discussed in the Additional file 1.

**Table 2:** Results of antibody measurement and seroprevalence

Antibody levels (Mean ± SD)	Patients with schizophrenia spectrum disorders (n = 100)*	IIH Controls (n = 39)*	p-value
**EBV**	n = 84 (84%)	n = 37 (95%)	Chi^2^ = 2.942 p = 0.086
Serum (U)	51,574.59 ± 50,164.67	54,007.71 ± 47,090.30	z = − 0.602 p = 0.547
CSF (U)	120.73 ± 155.75	79.02 ± 81.99	z = − 0.875 p = 0.382
CSF/Serum	2.81 ± 4.19	1.57 ± 0.84	z = − 2.768 **p = 0.006**
AI	0.93 ± 0.82	0.80 ± 0.29	z = − 0.535 p = 0.593
**CMV**	n = 41 (41%)	n = 15 (39%)	Chi^2^ = 0.075 p = 0.784
Serum (U)	27,638.94 ± 27,336.05	33,900.25 ± 33,022.25	z = − 0.916 p = 0.360
CSF (U)	64.14 ± 55.43	66.55 ± 77.03	z = − 0.139 p = 0.890
CSF/Serum	2.64 ± 1.05	2.16 ± 1.69	z = − 2.470 **p = 0.014**
AI	1.16 ± 0.47	1.11 ± 0.69	z = − 1.018 p = 0.309
**HSV1/2**	n = 59 (59%)	n = 26 (67%)	Chi^2^ = 0.694 p = 0.405
Serum (U)	62,392.74 ± 33,027.48	70,727.88 ± 44,320.81	z = − 0.019 p = 0.985
CSF (U)	160.16 ± 197.33	155.11 ± 130.48	z = − 0.048 p = 0.962
CSF/Serum	2.77 ± 3.93	2.17 ± 1.52	z = − 0.992 p = 0.321
AI	0.96 ± 0.45	1.01 ± 0.50	z = − 0.105 p = 0.916
**T. gondii**	n = 17 (17%)	n = 16 (41%)	Chi^2^ = 8.946 **p = 0.003**
Serum (U)	21,313.23 ± 13,672.51	27,006.46 ± 14,736.33	z = − 1.513 p = 0.136
CSF (U)	47.60 ± 42.40	64.54 ± 39.71	z = − 1.693 p = 0.094
CSF/Serum	2.31 ± 1.29	4.33 ± 6.46	z = − 0.036 p = 0.986
AI	0.86 ± 0.27	2. 91 ± 3.40	z = − 1.549 p = 0.127

Significant p-values are marked in bold. EBV Epstein–Barr virus

*U* unit, *CSF* cerebrospinal fluid, *CMV* human cytomegalovirus, *HSV* herpes simplex virus, *T. gondii* Txoxoplasma gondii, *SD* standard deviation, *IIH* idiopathic intracranial hypertension, *AI* antibody index

^*^Only measurable antibody levels were analyzed

### Routine cerebrospinal fluid diagnostics and neuronal autoantibody findings

In CSF routine diagnostics, the SSD group showed significantly higher total protein concentrations (p < 0.001) with significantly more patients with elevated protein levels than the control group (p = 0.010). AQs were also significantly elevated in patients with SSD (p < 0.001). After correction for sex in secondary analysis, the significant difference in total protein concentrations (F_(1,136)_ = 6.588, p = 0.011), number of patients with elevated total protein levels (β = 1.074, Wald = 4.625, p = 0.032), and AQ (F_(1,136)_ = 4.970, p = 0.027) persisted.

One patient presented with questionable anti-NMDA-R IgG autoantibodies in serum (negative in CSF; only tested in N = 74 patients). No autoantibodies against intracellular antigens could be found (N = 86; Additional file 2: Tables S2, S3).

### Instrumental diagnostics

Instrument-based diagnostics revealed MRI alterations in 63% of patients with SSD and EEG abnormalities in 31% of patients with SSD. Frequent abnormalities were non-specific white matter changes in MRI (28%) and intermittent generalized slowing in EEG (25%; see Additional file 2: Table S4).

### Correlation analyses

In the correlation analysis, EBV AI correlated significantly with disturbances of orientation (r = 0.253, p = 0.042; N = 65). HSV AI correlated with delusions (r = 0.363, p = 0.015; N = 44). For age, a negative correlation with CMV AI (r = − 0.383, p = 0.014; N = 41) could be detected. Negative correlations for Toxoplasma AI for ego-boundary disturbances (r = − 0.637, p = 0.026; N = 12) and hallucinations (r = − 0.826, p = 0.001; N = 12) could be found. EBV and HSV AI correlated with each other (r = 0.391, p = 0.004; N = 53).

### Characteristics of AI outliers and subanalyses

All outlier patients with AIs two standard deviations over the mean value are marked red in Fig. 1. Interestingly, when counting the borderline positive CMV AI in Patient 3 (Table 3), half of the patients with outlier AIs showed two elevated AIs, with Patient 1 presenting with two outlier AIs. In contrast, the CSF findings are all inconspicuous, except for oligoclonal bands (OCBs) in Patient 2, indicating an intrathecal oligoclonal IgG synthesis, and an unspecific identical OCB in CSF and serum in Patient 3, which is considered a normal finding, and may be due to the elevated AIs in these patients. Further diagnostic investigation with EEG and MRI identified only mild abnormalities in some patients. It is remarkable that the two outlier EBV patients both presented with intrathecal HSV1/2 antibody synthesis; in one of these patients, OCBs were also detected. These are indications of a polyspecific immune response rather than an acute EBV infection.

**Table 3:** Characteristics of outlier patients with increased pathogen specific AI

Patient	Sex	Age	Psychiatric syndrome	Somatic co-morbidity	AIs	Medication	CSF	Other immuno-logical changes	MRI	EEG	Previous inpatient stays	Course of disease
Pat. (1)	F	End 40	Schizoaffective syndrome ADHD	None	**EBV 6.01, HSV 2.53**, CMV/ T. gondii not measurable	Haloperidol, quetiapine, diazepam	Inconspicuous	None	Inconspicuous	Inconspicuous	Unknown	Chronic
Pat. (2)	M	Mid 20	Paranoid hallucinatory syndrome Depression	None	**EBV 5.61, HSV 1.74**, CMV/T. gondii not measurable	Olanzapine, escitalopram	Intrathecal oligoclonal IgG-synthesis (OCBs)	None	Inconspicuous	Inconspicuous	10	Chronic
Pat. (3)	M	Mid 20	Paranoid hallucinatory syndrome	None	**HSV 2.39, CMV 1.5**, T. gondii 0.68 EBV not measurable	Risperidone	1 identical oligoclonal band (OCB) in CSF and serum (non-specific)	None	Small developmental venous anomaly, otherwise inconspicuous	Inconspicuous	1	First diagnosis
Pat. (4)	F	End 40	Acute polymorphic psychotic disorder	Hyperthyroidism	**HSV 2.22,** EBV 0.98, CMV/T. gondii not measurable	Olanzapine	Inconspicuous	None	Inconspicuous	Inconspicuous	Unknown	Recurrent
Pat. (5)	F	Mid 20	Paranoid hallucinatory syndrome	Polycystic ovary syndrome, hyperandrogenemia	**CMV 2.66,** HSV 1.07, EBV 0.60, T. gondii not measurable	Clozapine, aripiprazole	Inconspicuous	None	Enlarged Virchow-Robin spaces	Inconspicuous	Unknown	Chronic
Pat. (6)	F	~ 20	Paranoid hallucinatory syndrome	None	**CMV 2.54**, EBV 1.38, HSV/T. gondii not measurable	Risperidone, promethazine	Inconspicuous	None	Pinealis cyst, small white matter lesion	Intermittend general slowing	None	First diagnosis

All elevated intrathecal pathogen-specific AIs are marked in bold

*F* female, *M* male, *BBB* blood–brain-barrier, *OCB* oligoclonal bands, *CSF* cerebrospinal fluid, *EBV* Epstein–Barr virus, *HSV* herpes simplex virus, *AI* antibody index

In a subgroup analysis of the 15 SSD patients with at least one elevated AI compared to the remaining 85 patients without elevated AI, no significant age or sex difference was found. The CSF protein concentrations were lower, on average, in the group of patients with elevated AI than in the rest of the SSD patients (total protein 351.20 vs. 472.14 mg/dl; AQ 4.37 vs. 5.77), but did not differ significantly. No significant differences in EEG and MRI findings were observed.

In a further subgroup analysis between patients with first-time SSD diagnosis (n = 42) and patients with chronic or relapsing SSD (n = 58), no significant age or sex difference was found. When assessing the specific AIs, a significantly higher mean EBV AI was observed in patients with chronic SSD relative to patients with first-time diagnosis (EBV AI: 1.10 ± 1.07 in 45 patients with chronic SSD vs 0.73 ± 0.25 in 39 patients with first-time diagnosis; z = − 2.933, p = 0.003). Similarly, when comparing the number of patients with elevated EBV AI values, there was a clear tendency of increased AIs towards the group with chronic SSD (elevated EBV AI > 1.5: 4 out of 45 with chronic SSD vs. 0 out of 39 with first-time diagnosis; Chi^2^ = 3.640, p = 0.056). No significant differences were observed for the other pathogens or the number of patients with multiple elevated AIs.

## Discussion

In this study, the difference between SSD patients and controls regarding intrathecal antibody synthesis from the specific pathogens EBV, CMV, HSV, and T. gondii was investigated, as an association with schizophrenia has been shown in previous studies [4, 59]. Nevertheless in this study, no significant difference was found between the two groups in the number of patients with elevated pathogen-specific AI or in the number of patients with multiple elevated AIs. Two patients with SSDs displayed polyclonal antibody synthesis with elevated AIs for EBV and HSV. Interestingly, there were higher EBV AI levels in the group of patients with chronic SSD compared to patients who were diagnosed with SSD for the first time.

### EBV

Of all pathogen-specific antibodies, those against EBV were the most frequently detected in the serum of patients and controls. This is not surprising, since EBV has a known high seroprevalence in healthy populations with 73–95%, and in patients with multiple sclerosis, seroprevalence is closer to 100% [1, 6, 18]. EBV infection in childhood was associated with the occurrence of a psychotic episode in adolescence in a prospective study [23]. In several previous studies, no differences in antibody levels of SSD patients with control populations have been observed [4, 59], whereas other studies reported elevated antibodies against the EBV VCA protein and whole virion with no differences in EBV EBNA-1 antibodies [11]. Elevated levels of EBV antibodies are associated, on the one hand, in combination with genetic susceptibility to schizophrenia with a greater risk for schizophrenia diagnosis [11], and on the other hand, with a lower level of cognitive performance [12]. The latter is of great interest, since in this study a correlation between the EBV-specific AI and the AMDP score for disturbances of orientation was observed. This may be a hint at greater cognitive impairment in SSD patients with an increased intrathecal synthesis of EBV antibodies. Nevertheless, controlled studies that account for the variety of confounding factors involving cognition are needed to reach a valid conclusion in this regard.

Furthermore, subanalysis revealed that elevated EBV AIs were associated with patients with chronic SSD compared to patients with first-time diagnoses independent of age and sex. There is little information available on EBV infections and the chronicity of SSDs. Although it is well-known that EBV causes latent infections in a wide variety of body regions, including the brain, with the potential to reactivate [12]. Such latent EBV infections and reactivations in the CNS have been associated with encephalitis in stem cell recipients [30] and inflammatory immune reactions in active multiple sclerosis lesions [6, 56]. With the potential for latent infections and recurrent reactivations in mind, it is imaginable that EBV may play a potential modulating role in a subgroup of patients with chronic SSD.

### CMV

The association of CMV and schizophrenia is much debated, and several contradictory results emerge [52]. Increased CMV antibody concentrations in serum [32, 45, 48, 50] and CSF [28] have been described in the literature in patients with schizophrenia; however, no significant differences have been reported in other studies [19, 25, 52, 53, 62] as in this study. The AI of CMV in this study was the only studied pathogen to correlate negatively with age. Thus, younger, possibly more immunocompetent patients, appear to be more prone to intrathecal CMV antibody synthesis. Albrecht et al. (1980) found no age difference regarding CMV antibody concentrations in serum, but observed an increase in the CSF/serum ratio of CMV antibodies along with increased age-dependent blood–brain-barrier permeability. Other antibody indices, such as from the John Cunningham virus in multiple sclerosis, tend to have a rather positive correlation while increasing with age [26].

### HSV

As with the other pathogens, the study evidence on HSV 1 and HSV 2 infections with schizophrenia is quite ambiguous [24, 28, 32, 33, 48, 53, 59]. Even clinically controlled trials of anti-infective therapy with valacyclovir were conducted, but failed to show symptom improvement [8]. However, there is strong evidence that serologic detection of HSV-1 affects cognitive functioning in patients with SSD [13, 55]. In particular, memory and attentional abilities seem to be impaired [55]. No evidence for this could be found in our study, but a correlation between HSV AI and delusions was observed. This is an interesting finding, as previous studies have described an association of HSV seropositivity with negative symptoms [7].

### Toxoplasmosis

A variety of studies have found an association between T. gondii infection and schizophrenia [28, 46, 51, 60], while others did not [24, 59, 60]. Among the latter one study reported that in areas with a low seroprevalence of T. gondii infections, patients with an established diagnosis of schizophrenia often have negative T. gondii findings, while patients with recent onset of psychosis might be associated with it [17, 60, 61]. Hence, the timing of toxoplasmosis infection with symptom onset of schizophreniform symptoms appears to play an important role [60]. In this study the proportion of patients with chronic SSD and first-time diagnosis is balanced; no difference between T. gondii AI could be found between these subgroups. Nevertheless, a very low seroprevalence of 17% was observed in SSD patients, which is in line with previously published negative studies. However, it is striking that the seroprevalence of the control group, with 43%, corresponds to the previously described seroprevalence of the general population in southern Germany with 44% [58]. A possible explanation may be the neuroleptic medication of the patient group, as inhibition of replication of T. gondii by neuroleptics has been shown in vitro [22]. Nevertheless, a previous study comparing T. gondii AIs from bipolar patients to patients with IIH reported no significant difference in the number of elevated AIs or seroprevalence [47]. Of the patients with SSD and available AIs, negative correlations were found for AMDP scores for ego (boundary) disturbances and hallucinations. In contrast, previous studies for T. gondii described seropositive patients with a higher intensity of psychotic symptoms [3].

### Polyspecific immune response

Two of the patients with chronic SSD and elevated EBV AIs also had intrathecal HSV1/2 antibody synthesis, with OCBs also detected in one of them, suggesting a polyspecific immune response. This is reminiscent of the MRZ reaction, which is positive in patients with multiple sclerosis and for which at least two elevated AIs of measles, rubella, and/or varicella zoster virus AIs have to be increased [21]. Among the cases with multiple elevated AIs detected here, patients with autoimmune psychosis could be hidden [35]. This phenomenon seems to be rare (at 2% in our cohort, 3% in the subgroup of chronic SSD). In line with this, an earlier study described a positive MRZ reaction in two of the 39 (5%) patients with SSDs [14]. Nevertheless, a similar rate of multiple positive AIs was found in our control group of IIH patients. The trigger of autoimmunity in these cases will have to be further investigated in the future.

## Limitations

Individual analysis of antibody concentrations in CSF and serum revealed significant higher CSF to serum ratios for EBV and CMV (Table 2). Nevertheless, when the pathogen-specific AIs were calculated with these quotients and the CSF/serum quotient of total IgG antibodies, no significant differences were observed between SSD and control group. This finding shows the importance of calculating AIs, instead of CSF/serum antibody quotients alone, to avoid wrong conclusions. A more detailed discussion of these methodological aspects can be found in the Additional file 1.

The major limiting factor is the neurological control group of patients with IIH, as the disorder may be associated with T. gondii [16, 36] or was also reported to be imitated by atypical HSV-2 infection [44]. Therefore, it could underestimate the study results. Furthermore, although only control patients with no known mental illness were included, no systematic screening for mental illness or subclinical psychiatric symptoms, as predescribed in IIH [34], was performed in the control group. For ethical reasons the establishment of a healthy control group with lumbar punctures was not possible.

Furthermore, a significant sex discrepancy may have influenced the results since, for example, an influence of sex on the seroprevalence of T. gondii is known [58]. Therefore, secondary analyses with correction for sex were conducted. Another influential factor may be the medication of the patient group. While antibody concentrations of CMV, for instance, were unchanged under medication in previous studies [54], other studies showed lower concentrations in treated patients compared to untreated patients [28]. In the case of T. gondii, there is evidence that neuroleptic medication in vitro may directly inhibit the replication of the protozoan and thus affect antibody concentrations [22]. In addition, potential influencing factors such as rural living or pet ownership (especially cats due to their relationship with T. gondii [58]) are not known. Regarding methodology, statements about absolute antibody concentrations are only possible to a limited extent since the ELISA kits were designed for the measurement of pathogen-specific AIs. Although ELISAs have improved significantly in recent years, some elaborate methods, such as reactivity to T. gondii proteins in the Western blot technique, appear even more sensitive in detecting seropositive patients [60]. Some Als were < 0.7 and thus could not be measured optimally (cf. Figure 1) as known from other studies [43, 47]. Finally, it must be mentioned that although the determination of pathogen-specific AIs entails many advantages, such as the detection of immunological responses in the CNS even years after pathogen contact or detection of polyspecific immune responses [47], it does not provide concrete information about infectious disease status or involvement.

## Conclusion

In summary, in this assessment of pathogen-specific intrathecal antibody synthesis by AI, we did not find significant group difference between SSD patients and the control group of IIH patients. Subanalyses of SSD patients revealed a possible role of latent EBV infection in chronic SSDs with evidence for a polyspecific immune response of the CNS in 3% of patient with chronic vs. 0% with first diagnosed SSD. Given the still highly inconclusive study situation, further investigations on the influence of infectious pathogens, especially EBV, on schizophrenia using multimodal approaches, including CSF analyses, are necessary.

## Supplementary Information

**Additional file 1: **Increased CSF/serum IgG ratios for EBV and CMV antibodies.

**Additional file 2: Table S1.** Clinical and demographic data. **Table S2.** Cerebrospinal fluid routine diagnostics. **Table S3.** Number of participants with abnormal cerebrospinal fluid diagnostics. **Table S4.** Number of magnetic resonance imaging (MRI) and electroencephalography (EEG) alterations in the patient group with schizophrenia spectrum disorders.

## Data Availability

All necessary information is displayed descriptively in the results section.
