# Supplementary material for: Antibody indices of infectious pathogens from serum and cerebrospinal fluid in patients with schizophrenia spectrum disorders
Source: Fluids Barriers CNS. 2022 Jul 29;19:61. doi: 10.1186/s12987-022-00355-7 (PMC9338642; doi:10.1186/s12987-022-00355-7)
Supplement: Supplementary file 1 — Additional file 1: Increased CSF/serum IgG ratios for EBV and CMV antibodies. [file 12987_2022_355_MOESM1_ESM.docx]

**Additional file 1**

**Increased CSF/serum IgG ratios for EBV and CMV antibodies**

The individual analysis of antibody concentrations in CSF and serum revealed significant higher CSF to serum ratios for EBV (p = 0.006) and CMV (p = 0.014; Table 2). Nevertheless, when with these quotients and the CSF/serum quotient of total IgG antibodies the pathogen-specific AIs were calculated, no significant differences were observed between SSD and control group.

For EBV, this effect appears to be based on the different levels of total IgG (2.98 in SSD patients vs. 1.98 in controls; z = -3.437, p = 0.001) rather than the pathogen-specific antibody itself. When the IgG index was calculated by dividing the IgG ratio and the albumin quotient (AQ) as a measure of autochthonous intrathecal IgG synthesis, no significant difference between the groups was observed (0.49 in SSD patients vs. 0.50 in controls; z = -1.785, p = 0.074). Similarly, this could be discussed for CMV with higher concentrations of total IgG in SSD patients (2.43 vs. 1.97; z = -1.610, p = 0.107) as well, although the group difference for total IgG was not significant. Furthermore, in secondary analysis with correction for sex, a trend could still be detected for higher CSF to serum ratios in EBV (F_(1,118) =_3.082, p = 0.082); but no longer for CMV (F_(1,53) =_0.935, p = 0.338).

To explore the relationship between infectious pathogens and schizophrenia, various methods, such as large Scandinavian registry studies [1, 2], direct antigen detection [3], or antibody concentrations in serum and CSF [4, 5] have been used. Due to the proximity to the brain, examinations of CSF play a special role in this regard. For instance, AI is capable of providing information about brain inflammation through intrathecal immunoglobulin synthesis, which has been traceable for many years when compared to the acute infection [6, 7]. Nevertheless, most studies refer to antibody concentrations in serum, since they are more easily obtainable. In this study, no significant differences in the serum antibody concentrations for EBV, CMV, HSV1/2, and T. gondii could be found in line with some previous studies [8, 9], while other investigations report contrastingly elevated levels [10, 11]. Similar to CSF, no significant differences in antibody concentrations were observed as previously described [12, 13], while other studies found elevated levels [13, 14]. These immunoglobulin concentrations in serum and CSF vary between individuals and with age; therefore, for an individual patient, a statement about pathogenicity based on the concentration level is not possible [15]. Furthermore, IgG antibodies only indicate some degree of contact with a pathogen, without an acute inflammation having occurred. To assess the affection of the central nervous system, intrathecal antibody synthesis is of interest [16]. For this purpose, a quotient can be formed from CSF and serum. Thus, Albrecht et al. (1980) suspected an intrathecal synthesis in the case of an increased CMV antibody quotient in patients with schizophrenia compared to healthy controls [14]. This could also be suspected in this study with significantly increased CSF/serum ratios of EBV and CMV antibodies compared to the healthy control group. However, this ignores the important issue that the effect may not be based on an increase in a pathogen-specific antibody concentration, but on an overall increase in immunoglobulins in CSF [17]. Indeed, in the present study, at least for all SSD patients with antibodies against EBV, an increased total IgG quotient could be shown compared to controls. In patients with schizophrenia, increased IgG concentrations in the CSF and elevated CSF-to-serum total IgG ratios have previously been described in the literature [18, 19]. However, the immunoglobulins are not the only proteins elevated in CSF: total protein and albumin quotients are also significantly increased in comparison to the controls, which is an already well-known phenomenon [5, 20]. The AQ is the gold standard for evaluating the BCSFB and indicates, with elevated levels, a dysfunction of the BCSFB or a disturbed CSF flow resulting in protein accumulation in the CSF [20, 21]. However, how can we distinguish between intrathecally produced antibodies and blood-derived antibodies due to BCSFB leakage or decreased CSF drain? For total IgG, the IgG index can be calculated by dividing the IgG quotient by the AQ. When elevated, the IgG index indicates intrathecal immunoglobulin synthesis. Interestingly, decreases to normal IgG indices have been reported in schizophreniform psychosis [4, 5], which fits our study with a tendency to decrease IgG indices compared to controls. However, it is important to note that one patient with clear intrathecal oligoclonal IgG synthesis was detected in the patient group and none in the control group, despite the fact that this difference is not significant in agreement with previous studies [23]. For the assessment of the intrathecal synthesis of specific antibodies, the previously mentioned specific AI should be calculated [17]. In this study, no significant differences in specific antibodies for EBV or CMV could be observed when the specific antibody indices were used for comparison.

**References**

1. Mortensen PB, Pedersen CB, Hougaard DM, et al. A Danish National Birth Cohort study of maternal HSV-2 antibodies as a risk factor for schizophrenia in their offspring. *Schizophrenia Research*. 2010;122(1-3):257-263. Published July 2, 2010.

2. Benros ME, Nielsen PR, Nordentoft M, Eaton WW, Dalton SO, Mortensen PB. Autoimmune diseases and severe infections as risk factors for schizophrenia: a 30-year population-based register study. *The American journal of psychiatry*. 2011;168(12):1303-1310.

3. Cinque P, Cleator GM, Weber T, Monteyne P, Sindic CJ, van Loon AM. The role of laboratory investigation in the diagnosis and management of patients with suspected herpes simplex encephalitis: a consensus report. The EU Concerted Action on Virus Meningitis and Encephalitis. *Journal of neurology, neurosurgery, and psychiatry*. 1996;61(4):339-345.

4. Melkersson K, Bensing S. Signs of impaired blood-brain barrier function and lower IgG synthesis within the central nervous system in patients with schizophrenia or related psychosis, compared to that in controls. *Neuro endocrinology letters*. 2018;39(1):33-42.

5. Orlovska-Waast S, Köhler-Forsberg O, Brix SW, et al. Cerebrospinal fluid markers of inflammation and infections in schizophrenia and affective disorders: A systematic review and meta-analysis. *Molecular psychiatry*. 2019;24(6):869-887. https://www.nature.com/articles/s41380-018-0220-4.pdf. Accessed November 17, 2019.

6. Reiber H, Peter JB. Cerebrospinal fluid analysis: disease-related data patterns and evaluation programs. *Journal of the neurological sciences*. 2001;184(2):101-122.

7. Stich O, Andres TA, Gross CM, Gerber SI, Rauer S, Langosch JM. An observational study of inflammation in the central nervous system in patients with bipolar disorder. *Bipolar disorders*. 2015;17(3):291-302.

8. Delisi LE, Smith SB, Hamovit JR, et al. Herpes simplex virus, cytomegalovirus and Epstein-Barr virus antibody titres in sera from schizophrenic patients. *Psychological medicine*. 1986;16(4):757-763.

9. Witte LD de, van Mierlo HC, Litjens M, Klein HC, Bahn S, Osterhaus AD. The association between antibodies to neurotropic pathogens and schizophrenia: a case-control study. *NPJ schizophrenia*. 2015;1:15041. https://pubmed.ncbi.nlm.nih.gov/27336045/. Published November 4, 2015.

10. Tedla Y, Shibre T, Ali O, et al. Serum antibodies to Toxoplasma gondii and Herpesvidae family viruses in individuals with schizophrenia and bipolar disorder: a case-control study. *Ethiopian medical journal*. 2011;49(3):211-220.

11. Mohagheghi M, Eftekharian MM, Taheri M, Alikhani MY. Determining the IgM and IgG antibodies titer against HSV1, HSV2 and CMV in the serum of schizophrenia patients. *Human antibodies*. 2018;26(2):87-93.

12. King DJ, Cooper SJ, Earle JA, Martin SJ, McFerran NV, Wisdom GB. Serum and CSF antibody titres to seven common viruses in schizophrenic patients. *The British journal of psychiatry : the journal of mental science*. 1985;147:145-149.

13. Leweke FM, Gerth CW, Koethe D, et al. Antibodies to infectious agents in individuals with recent onset schizophrenia. *European archives of psychiatry and clinical neuroscience*. 2004;254(1):4-8.

14. Albrecht P, Boone E, Fuller Torrey E, Hicks J, Daniel N. Raised cytomegalovirus-antibody level in cerebrospinal fluid of schizophrenic patients. *The Lancet*. 1980;316(8198):769-772.

15. Jazayeri MH, Pourfathollah AA, Rasaee MJ, Porpak Z, Jafari ME. The concentration of total serum IgG and IgM in sera of healthy individuals varies at different age intervals. *Biomedicine & Aging Pathology*. 2013;3(4):241-245.

16. Stich O, Jarius S, Kleer B, Rasiah C, Voltz R, Rauer S. Specific antibody index in cerebrospinal fluid from patients with central and peripheral paraneoplastic neurological syndromes. *Journal of neuroimmunology*. 2007;183(1-2):220-224. Published January 16, 2007.

17. Reiber H, Lange P. Quantification of virus-specific antibodies in cerebrospinal fluid and serum: sensitive and specific detection of antibody synthesis in brain. *Clinical chemistry*. 1991;37(7):1153-1160.

18. Müller N. Immunoglobulin and albumin content of cerebrospinal fluid in schizophrenic patients: Relationship to negative symptomatology. *Schizophrenia Research*. 1995;14(3):223-228.

19. Severance EG, Gressitt KL, Alaedini A, et al. IgG dynamics of dietary antigens point to cerebrospinal fluid barrier or flow dysfunction in first-episode schizophrenia. *Brain, behavior, and immunity*. 2015;44:148-158. Published September 20, 2014.

20. Endres D, Meixensberger S, Dersch R, et al. Cerebrospinal fluid, antineuronal autoantibody, EEG, and MRI findings from 992 patients with schizophreniform and affective psychosis. *Translational Psychiatry*. 2020;10(1):279. Published August 12, 2020.

21. Reiber H. Flow rate of cerebrospinal fluid (CSF) — A concept common to normal blood-CSF barrier function and to dysfunction in neurological diseases. *Journal of the neurological sciences*. 1994;122(2):189-203.

22. Asgari M, Zélicourt DA de, Kurtcuoglu V. Barrier dysfunction or drainage reduction: Differentiating causes of CSF protein increase. *Fluids and barriers of the CNS*. 2017;14(1):14.

23. Roos RP, Davis K, Meltzer HY. Immunoglobulin studies in patients with psychiatric diseases. *Archives of general psychiatry*. 1985;42(2):124-128.
