# Supplementary material for: Antibody indices of infectious pathogens from serum and cerebrospinal fluid in patients with schizophrenia spectrum disorders
Source: Fluids Barriers CNS. 2022 Jul 29;19:61. doi: 10.1186/s12987-022-00355-7 (PMC9338642; doi:10.1186/s12987-022-00355-7)
Supplement: Supplementary file 2 — Additional file 2: Table S1. Clinical and demographic data. Table S2. Cerebrospinal fluid routine diagnostics. Table S3. Number of participants with abnormal cerebrospinal fluid diagnostics. Table S4. Number of magnetic resonance imaging (MRI) and electroencephalography (EEG) alterations in the patient group with schizophrenia spectrum disorders. [file 12987_2022_355_MOESM2_ESM.docx]

**Additional file 2:**

**Tables**

|  | **SSD Patients**  **(N = 100)** | **IIH controls (N = 39)** | **Statistics** |
| --- | --- | --- | --- |
| **Sex** | 40 M : 60 F | 6 M : 33 F | Chi^2 =^7.678  **p = 0.006** |
| **Average age ± SD** (age range) | 33.7 ± 12.0  (18-65 years) | 34.6 ± 12.0  (18-61 years) | z = -0.516  p = 0.606 |
| **Diagnoses**  Paranoid schizophrenia [F20.0]  Hebephrenic schizophrenia [F20.1]  Catatonic schizophrenia [F20.2]  Delusional Disorder [F22.0]  Acute polymorphic psychotic [F23.1]  Schizoaffective disorder [F25.X]  -  **Course of disease**  First-time diagnosis  Chronic/Recurrent | 56 (56%)  2 (2%)  2 (2%)  6 (6%)  4 (4%)  30 (30%)  42 (42%)  58 (58%) |  |  |
| **Psychiatric comorbidity**  Depression  ADHD  Autism spectrum disorder  Other Developmental disorder  OCD  Borderline personality disorder  PTSD  Anorexia nervosa in youth  Substance dependence in history | 2 (2%)  6* (6%)  1 (1%)  2 (2%)  3 (3%)  1 (1%)  1 (1%)  1 (1%)  3 (3%) |  |  |
| **Neurologic comorbidity**  Seizures  Concussion  Migraine  Vertigo  Other  Overall | 2** (2%)  5*** (5%)  1 (1%)  1 (1%)  1**** (1%)  10(10%) |  |  |
| **Psychotropic drugs at the time of sampling**  SSRI  SSNRI  Mirtazapine  Tricyclic antidepressants  “Typical neuroleptics” with high-potency  “Typical neuroleptics” with low-potency  “Atypical neuroleptics”  Lithium  Benzodiazepines  Anticonvulsants  Unmedicated | 7 (7%)  5 (5%)  3 (3%)  2 (2%)  6 (6%)  10 (10%)  96 (96%)  10 (10%)  16 (16%)  15 (15%)  3 (3%) | -  -  -  -  -  -  -  -  -  -  - |  |
| **Civil status**  Single  Married  Divorced  Widowed  Unknown  **Educational Level**  Low  Intermediate  High  Unknown | 67 (67%)  15 (15%)  5 (5%)  1 (1%)  12 (12%)  18 (18%)  23 (23%)  46 (46%)  13 (13%) |  |  |
| **Employment**  Unemployed  Working  In training  Disability Retirement  Scheduled Retirement  Housewife/-man  Unknown  **Living situation**  Alone  With partner/family  With parents/custodian  Others  Unknown  **Family history for any psychiatric disease*******  Positive  Negative  Unknown  **Number of previous inpatient stays**  None  1  2  3  More than 3  Unknown  **Number of suicide attempts**  None  1  2  More than 2  Unknown | 18 (18%)  27 (27%)  28 (28%)  8 (8%)  1 (1%)  6 (6%)  12 (12%)  34 (34%)  23 (23%)  27 (27%)  5 (5%)  11 (11%)  42 (42%)  43 (43%)  15 (15%)  35 (35%)  12 (12%)  10 (10%)  9 (9%)  20 (20%)  14 (14%)  65 (65%)  8 (8%)  9 (9%)  3 (3%)  15 (15%) |  |  |

**Table S1: Clinical and demographic data**. *Of which one only in childhood **One patient with drug-induced (>10 years ago) and one with two unclear generalized seizures. ***One 7 years before lumbar puncture and two in childhood ****Cervical Radiculopathy C8. *****In first-degree relatives. Abbreviations: IIH = Idiopathic intracranial hypertension, F = female, M = male, SD = standard deviation, OCD = obsessive-compulsive disorder, PTSD = posttraumatic stress disorder, CSF = cerebrospinal fluid, cMRI = cerebral magnetic resonance imaging, EEG = electroencephalography, SSRI = selective serotonin reuptake inhibitor, SSNRI = selective serotonin/noradrenaline reuptake inhibitor.

|  | **Reference**  (Hufschmidt et al., 2017) | **SSD patients (N = 100)** | **IIH controls (N = 39)** | **Statistics** |
| --- | --- | --- | --- | --- |
| **White blood cell count**  (Mean = SD) | < 5/µl | 1.73 ± 1.52 | 2.60 ± 7.59* | z = -1.017 p = 0.309 |
| **Total protein**  (Mean = SD) | < 450mg/l | 454.00 ± 302.10 | 309.33 ± 142.53 | z = -4.022 **p < 0.001** |
| **Albumin quotient**  (Mean = SD) | < 40y.: 6.5 x 10^-3^;  40-60y.: 8 x 10^-3^;  >60y.: 9.3 x 10^-3^) | 5.56 ± 4.29 | 3.93 ± 1.81 | z = -3.773 **p < 0.001** |
| **IgG Index**  (Mean = SD) | ≤0.7 mg/l | 0.49 ± 0.06 | 0.50 ± 0.04 | z = -1.928 p = 0.054 |

**Table S2: Cerebrospinal fluid routine diagnostics.** Abbreviations: SSD = schizophrenia spectrum disorder, IIH = Idiopathic intracranial hypertension, SD = standard deviation, IgG = immunoglobulin G, N = number. *Data of only 35 controls are available. One of them suffered from reactive pleocytosis (46 cells/µl), which regressed independently to normal white blood cell counts. The magnetic resonance imaging was normal in this patient. No other cause for the transient, self-limiting pleocytosis was detected in this patient.

| Number of patients with increased CSF parameters (reference (Hufschmidt et al., 2017) | **SSD patients (N = 100)** | **IIH controls (N = 39)** | **Statistics** |
| --- | --- | --- | --- |
| **Increased white blood cell counts** (≥5/µl) | 5 (5%) | 1 (2.9%)* | Chi^2 =^0.280  p = 0.596 |
| **Increased total protein** (>450mg/l) | 38 (38%) | 6 (15.4%) | Chi^2 =^6.633  **p = 0.010** |
| **Increased age-dependent albumin quotient**  (<40y.: 6.5 x 10^-3^  40-60y.: 8 x 10^-3^  >60y.: 9.3 x 10^-3^) | 16 (16%) | 2 (5.1%) | Chi^2 =^2.942  p = 0.086 |
| **Increased IgG index** (>0.7 mg/l) | 1 (1%) | 0 (0%) | Chi^2 =^0.393  p = 0.531 |
| **CSF specific OCBs** | 1** (1%) | 0*** (0%) | Chi^2 =^2.409  p = 0.531 |

**Table S3: Number of participants with abnormal cerebrospinal fluid diagnostics.** SSD = schizophrenia spectrum disorder, IIH = Idiopathic intracranial hypertension, WBC = white blood cell, SD = standard deviation, IgG = immunoglobulin G, OCBs = oligoclonal bands, CSF = cerebrospinal fluid, N = number. *Only data of 35 controls are available. One of them suffered from reactive pleocytosis (46 cells/µl) due to repetitive lumbar punctures, which regressed independently to normal WBC counts in the follow-up lumbar puncture. The magnetic resonance imaging was normal in this patient. No other cause for the transient, self-limiting pleocytosis was detected in this patient. **Five findings were not considered positive: Two patients had some weak identical bands in CSF and serum as well as three patients had isolated OCBs in the CSF. ***Only data of 38 controls was available. Three patients had an isolated OCB which were not considered positive.

| **MRI alterations** | **Patients (N = 100)** |
| --- | --- |
| White matter alterations/cerebral microangiopathy | 28 (28%) |
| Post inflammatory changes | 5 (5%) |
| Tumors | 2* (2%) |
| Atrophic changes | 7 (7%) |
| Cysts | 10 (10%) |
| Asymmetry of the ventricular system | 5 (5%) |
| Anatomical variants | 8 (8%) |
| Other | 2****** (2%) |
| **Patients with overall MRI alterations** | 63/100 patients (63%) |
| **EEG alterations** | **Patients (N = 100)** |
| Continuous generalized/ regional slow activity | 0 (0%) |
| Intermittent generalized slow activity | 25 (25%) |
| Intermittent focal slow activity | 5(5%) |
| Epileptiform discharges | 4 (4%) |
| **Total number of EEG alterations** | 31/100 patients (31%) |

**Table S4: Number of magnetic resonance imaging (MRI) and electroencephalography (EEG) alterations in the patient group with schizophrenia spectrum disorders.** *Small meningioma and adenohypophyseal microadenoma. **Subtle subarachnoid findings with possible inflammation and enlarged pituitary gland. Abbreviations: MRI = magnetic resonance imaging, EEG = electroencephalography, N = number.
